# Introducing BASE: the Biomes of Australian Soil Environments soil microbial diversity database

**DOI:** 10.1186/s13742-016-0126-5

**Published:** 2016-05-18

**Authors:** Andrew Bissett, Anna Fitzgerald, Thys Meintjes, Pauline M. Mele, Frank Reith, Paul G. Dennis, Martin F. Breed, Belinda Brown, Mark V. Brown, Joel Brugger, Margaret Byrne, Stefan Caddy-Retalic, Bernie Carmody, David J. Coates, Carolina Correa, Belinda C. Ferrari, Vadakattu V. S. R. Gupta, Kelly Hamonts, Asha Haslem, Philip Hugenholtz, Mirko Karan, Jason Koval, Andrew J. Lowe, Stuart Macdonald, Leanne McGrath, David Martin, Matt Morgan, Kristin I. North, Chanyarat Paungfoo-Lonhienne, Elise Pendall, Lori Phillips, Rebecca Pirzl, Jeff R. Powell, Mark A. Ragan, Susanne Schmidt, Nicole Seymour, Ian Snape, John R. Stephen, Matthew Stevens, Matt Tinning, Kristen Williams, Yun Kit Yeoh, Carla M. Zammit, Andrew Young

**Affiliations:** CSIRO, Oceans and Atmosphere, Hobart, Tasmania Australia; Bioplatforms Australia, Sydney, New South Wales Australia; Centre for Comparative Genomics, Murdoch University, Perth, Western Australia Australia; Victorian Department of Economic Development, Jobs, Transport and Resources and La Trobe University, Agribio Centre, Bundoora, Victoria 3083 Australia; CSIRO Land and Water, Adelaide, South Australia Australia; School of Biological Sciences and the Environment Institute, University of Adelaide, North Terrace Adelaide, South Australia 5005 Australia; School of Agriculture and Food Science, The University of Queensland, St Lucia, Queensland 4072 Australia; Parks Australia, Department of the Environment, Canberra, ACT 2601 Australia; School of Biotechnology and Biomolecular Sciences, UNSW Australia, Sydney, New South Wales 2052 Australia; School of Earth, Atmosphere and Environment, Monash University, Clayton, Victoria 3800 Australia; Science and Conservation Division, Department of Parks and Wildlife, Perth, Western Australia Australia; DEDJTR Rutherglen, Melbourne, Victoria Australia; Ramaciotti Centre for Genomics, University of New South Wales, Sydney, New South Wales Australia; School of Biotechnology and Biomolecular Sciences, University of New South Wales, Sydney, New South Wales 2052 Australia; CSIRO Agriculture, Adelaide, South Australia 5064 Australia; CSIRO, National Research Collections Australia, Canberra, Australian Capital Territory Australia; Hawkesbury Institute for the Environment, Western Sydney University, Penrith, New South Wales Australia; Australian Genome Research Facility Ltd, Walter and Eliza Hall Institute, Parkville, Victoria Australia; Australian Centre for Ecogenomics, School of Chemistry and Molecular Biosciences, The University of Queensland, St Lucia, Queensland 4072 Australia; Institute for Molecular Bioscience, The University of Queensland, St Lucia, Queensland 4072 Australia; Australian SuperSite Network, James Cook University, Townsville, Queensland Australia; University of Tasmania, Hobart, Tasmania Australia; Australian Genome Research Facility Ltd, Adelaide, South Australia Australia; Atlas of Living Australia, CSIRO, Canberra, Australian Capital Territory Australia; CSIRO Land and Water, Canberra, ACT Australia; Agriculture and Agri-food Canada, Science and Technology branch, 2585 County Road 20, Harrow, ON N0R 1G0 Canada; Department of Agriculture and Fisheries, Brisbane, Queensland Australia; Australian Antarctic Division, Department of Sustainability, Environment, Water, Population and Communities, 203 Channel Highway, Kingston, Tasmania 7050 Australia; University of Queensland, Earth Sciences, St Lucia, Brisbane, Queensland 4072 Australia

**Keywords:** Microbiology, Microbial ecology, Soil biology, Australia, Database, Microbial diversity, Metagenomics

## Abstract

**Background:**

Microbial inhabitants of soils are important to ecosystem and planetary functions, yet there are large gaps in our knowledge of their diversity and ecology. The ‘Biomes of Australian Soil Environments’ (BASE) project has generated a database of microbial diversity with associated metadata across extensive environmental gradients at continental scale. As the characterisation of microbes rapidly expands, the BASE database provides an evolving platform for interrogating and integrating microbial diversity and function.

**Findings:**

BASE currently provides amplicon sequences and associated contextual data for over 900 sites encompassing all Australian states and territories, a wide variety of bioregions, vegetation and land-use types. Amplicons target bacteria, archaea and general and fungal-specific eukaryotes. The growing database will soon include metagenomics data. Data are provided in both raw sequence (FASTQ) and analysed OTU table formats and are accessed via the project’s data portal, which provides a user-friendly search tool to quickly identify samples of interest. Processed data can be visually interrogated and intersected with other Australian diversity and environmental data using tools developed by the ‘Atlas of Living Australia’.

**Conclusions:**

Developed within an open data framework, the BASE project is the first Australian soil microbial diversity database. The database will grow and link to other global efforts to explore microbial, plant, animal, and marine biodiversity. Its design and open access nature ensures that BASE will evolve as a valuable tool for documenting an often overlooked component of biodiversity and the many microbe-driven processes that are essential to sustain soil function and ecosystem services.

## Data description

Human society is dependent on the ecosystem goods and services mediated by soil organisms [1]. Soils filter water, provide the growth medium for vegetation and crops, mediate global carbon and nutrient cycles, degrade xenobiotics, and are habitats for many organisms. Soils are a valuable source of biologically active industrial and medical compounds, are a storage and remediation medium for waste, and are sources for mineral exploration. The resident microbial communities mediate most soil processes, yet we know comparatively little about their diversity, biogeography, community assembly and evolutionary processes, symbiotic networks, adaptation to environmental gradients, temporal stability or responses to perturbation [2, 3]. Critically, the relationship between microbial identity and abundance (community composition), species interactions (community structure) and biogeochemical rate transformations (bioactivity) in natural and domesticated soils are largely unknown, which limits our influence on these factors to maximise desirable outcomes. This knowledge gap is at odds with observations that microbial communities make substantial contributions to ecosystem processes, as demonstrated in simple microcosms [4, 5] and in natural ecosystems [6–9]. Better understanding of soil-related microbial communities and processes is required to ensure continued (or improved) provision of the soil-moderated ecosystem services that promote environmental and human health, food security, mineral wealth and climate stability.

**Fig. 1:**
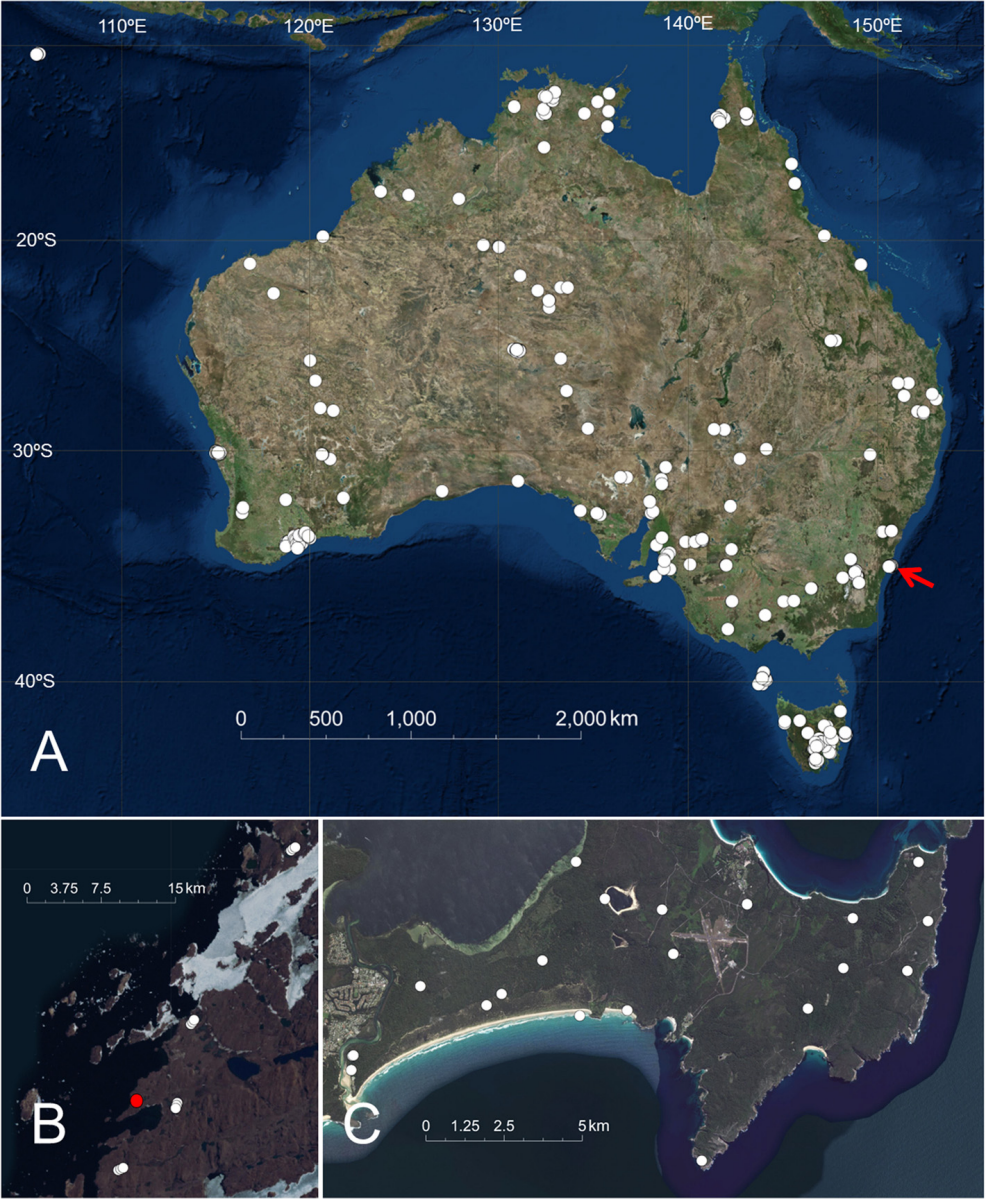
Position of BASE sample sites (August 2015). **a** Australian mainland and Christmas Island samples; **b** location of Antarctic sampling locations (white), with Davis station indicated in red; and **c** finer detail of sampling position indicated by red arrow in (**a**)

Most soil microorganisms cannot be cultured using standard microbial growth media [10]. Many were unknown until the 1990s when phylogenetic marker gene sequencing (meta-barcoding) revealed that they constitute the most diverse microbial communities on Earth [11]. DNA shotgun sequencing of environmental samples (metagenomics) soon revealed that microbial taxonomic diversity was also reflected in the richness of functional genes and pathways encoded in their genomes [12]. Only recently, however, have advances in high-throughput sequencing and bioinformatics made it possible to obtain data sets that are commensurate with the complexity of microbial communities. Nonetheless, to do this on a scale enabling generalised conceptual advances in ecological understanding, rather than in a smaller, piecemeal manner, requires targeted, coordinated and highly collaborative efforts. The Biomes of Australian Soil Environments (BASE) project (http://www.Bioplatforms.Com/soil-biodiversity/) is one such effort. BASE now provides a database of amplicon data (with metagenomic data currently being generated), complete with rich contextual information on edaphic, aboveground diversity and climate. These data were collected according to stringent guidelines across the Australian continent and extending into Antarctica (Fig. 1, Table 1). This database provides researchers with a national framework data set of microbial biodiversity encompassing much of the soil, vegetation and climate variation within Australia, and is set in the context of a cultural progression in science towards open access to data [13]. The BASE database represents infrastructure that can, among other things, be used to investigate the evolution of Australian soil microbes; biogeographic patterns of microbial community change and their environmental drivers; effects of land management on genes, functions, species or community assemblages; use as indicators for underlying mineral deposits and restoring degraded environments. With many soils in Australia (and globally) considered severely degraded, efforts to restore the soil physical and chemical properties of soil must be complemented with restoring biological function. BASE data will support efforts to manage soil microbes for improved ecological and agricultural outcomes, just as microbial medicine has developed into a potent tool to promote human health.

**Table 1:** Contextual data collected from each soil sample

Soil chemical properties		
moisture	Total Carbon	Zinc
Ammonium	Organic Carbon	Exchangeable Aluminium
Nitrate	Conductivity	Exchangeable Calcium
Total Nitrogen	pH	Exchangeable Magnesium
Phosphorus	Copper	Exchangeable Potassium
Potassium	Iron	Sodium
Sulphur	Manganese	Boron
Soil physical properties		
Texture	Color	Particle size distribution
Soil/site descriptors		
Overlying vegetation identity	Aspect	Elevation
Slope	Landscape position	Land-use history
Land-use Management		

## Selection and characteristics of soil samples

As of August 2015 the BASE data set represents >1400 samples taken from 902 locations across Australia (Fig. 1). These samples represent a wide variety of Australian bioregions and land-uses, and were collected from the soil inhabited by a diverse array of plant communities. Samples span a continental scale (>7.7 million km^2^).

To investigate microbial diversity in soils, each sample was subjected to phylogenetic marker (amplicon) sequencing to characterise the diversity of bacterial (16S rRNA gene), archaeal (16S rRNA gene) and eukaryotic (18S rRNA gene) community assemblages. Fungal diversity was captured to a certain extent by the 18S rRNA gene amplicon; however, because fungi are such an important component of soils, and because the internal transcribed spacer (ITS) region is more informative than 18S rRNA for many fungal groups, we also included a fungal-specific ITS region amplicon to characterise fungal community assemblages. These amplicons cover the diverse range of microbes resident in soils.

## Methods

**Fig. 2:**
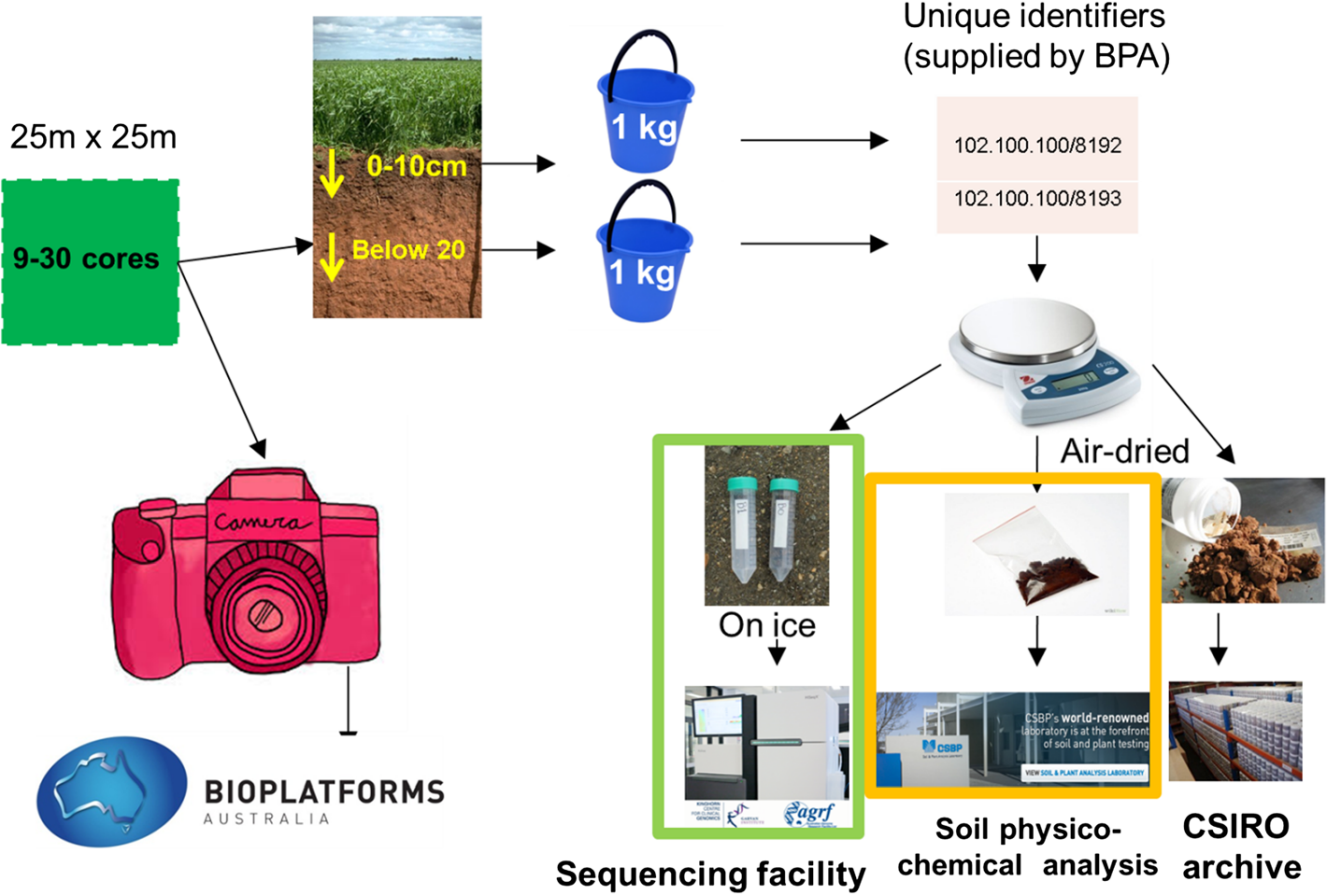
Sampling strategy. Approximately 1 kg of soil was taken, at two soil depths, by bulking 9 – 30 soil cores a 25 × 25 m quadrat. Each sample was assigned a unique identifier and subdivided for DNA extraction and sequencing, soil physico-chemical analyses and soil and DNA sample archiving for future use. A photograph of each site was also taken

Data collection followed the conceptual outline given in Fig. 2.

### Soil sampling

Soil samples were collected from 902 sites across Australia (Fig. 1) according to the methods described at the BASE data portal (Http://www.Bioplatforms.Com/sample-collection-procedure). These sites covered 27 IBRA 7 regions (Interim Biogeographic Regionalisation for Australia (https://www.Environment.Gov.Au/land/nrs/science/ibra#ibra). Many land-use categories were covered, representing most key vegetation types, and about 50 % of samples came from conservation reserves. Native restoration sites and production landscapes, including orchards and cereal croplands, were also sampled. Briefly, each mainland Australian soil sample comprised nine discrete soil samples from a 25 × 25 m quadrat sampled at two depth ranges (0–0.1 and 0.2–0.3 m), while Antarctic samples comprised the 0–0.1 m horizon only. Two discontinuous depths (0–0.1 m and 0.2–0.3 m) were sampled to ensure independent samples from both surface and shallow subsurface. Eight samples were taken at the corners and mid-points of the 25 × 25 m sides of the quadrat, and one from the centre. The quadrat size was chosen to represent the smallest pixel size of Australian soil mapping efforts [14] and to ensure enough soil for sequencing, chemical/physical analyses and sample archiving. While the 25 × 25 m sample unit size does not allow questions of finer scale (<25 m) heterogeneity to be addressed, it does allow high level integration with current Australian soil [15] and aboveground diversity mapping efforts [16], and facilitates meaningful temporal sampling (single point sampling is destructive and so not amenable to temporal sampling efforts). The nine subsamples were combined for each depth, to return a single surface and deeper soil sample per quadrat. Samples for molecular analysis were stored on ice until they could be frozen and transported to either the Adelaide node of the Australian Genome Research Facility (AGRF) laboratories (Australian samples) or, for the Antarctic samples, the Australian Antarctic Division (AAD), for DNA extraction. Australian samples for chemical and physical analysis were air-dried and transported to CSBP Laboratories (Perth, Western Australia) (https://www.Environment.Gov.Au/land/nrs/science/ibra#ibra), while edaphic properties of Antarctic samples were determined by the AAD. To minimise operator bias DNA extraction was carried out at AGRF or AAD (Antarctic samples only). At the time of sampling all other contextual data were collected including: sample location (coordinates taken at the centre point of the sampling quadrat), overlying plant cover (coverage and composition), slope, elevation above sea level, position in landscape (upper, mid, lower slope, valley, ridge) and land-use history.

### Contextual data

Soil chemical and physical attributes were usually determined at CSBP Laboratories. Soil moisture (% GWC) was measured gravimetrically [17], and ammonium and nitrate levels were determined colorometrically, following extraction with 1 M potassium chloride (25 °C) [18, 19]. Available phosphorus and potassium were measured using the Colwell method [17]. Sulphur levels were determined by the Blair/Lefroy Extractable Sulphur method [20]. Organic carbon was determined using the Walkley-Black method [21]. For pH analysis, CaCl pH and electrical conductivity (EC_1:5_), soils were extracted in deionised water for 1 h to achieve a soil:solution ratio of 1:5. The water pH and EC_1:5_ of the extract were subsequently measured using a combination pH electrode; calcium chloride solution was then added to the soil solution and, after thorough mixing, the calcium chloride pH determined [17]. Diethylene-triamine-pentaacetic acid (DTPA) extractable trace elements (Cu, Fe, Mn, Zn) were determined by atomic absorption spectroscopy following extraction with (DPTA) for 2 h [17]. Soils were extracted with a 0.01 M calcium chloride solution and analysed for extractable aluminium using inductively coupled plasma spectroscopy (ICP) [22]. Boron was measured by ICP after hot CaCl_2_ extraction [17]. Soil exchangeable cations (Mg, K, Na, Ca) were determined using a 1:5 soil:water extraction. This test was used in combination with the NH_4_Cl_2_/BaCl_2_ extractable exchangeable cations test, where the value for water soluble exchangeable cations is subtracted from the value for NH_4_Cl_2_/BaCl_2_ extractable exchangeable cations [17].

Soil particle size distribution was also measured. Soils were sieved to 2 mm (particles greater than 2 mm were considered gravel), treated with hydrogen peroxide to remove organic matter, and then treated with a 1:1 calgon–sodium hydroxide mixture to disperse particles. Using a standardised table of particle sedimentation times, 25 ml aliquots were removed from the shaken sample and the remaining sample sieved. The samples were evaporated, oven-dried and weighed to determine the sand, silt and clay contents [23].

### DNA extraction

All soil DNA was extracted in triplicate according to the methods employed by the Earth Microbiome Project (Http://www.Earthmicrobiome.Org/emp-standard-protocols/dna-extraction-protocol/).

### Sequencing

Sequencing was carried out using an Illumina MiSEQ, as described in detail both on the BASE protocols webpage (Https://ccgapps.Com.Au/bpa-metadata/base/information) and in the sequencing_methods_readme.txt on the data portal. Briefly, amplicons targeting the bacterial 16S rRNA gene (27 F–519R; [24, 25]), archaeal 16S rRNA gene (A2F–519R; [25, 26]), fungal ITS region (ITS1F–ITS4 [27, 28]) and eukaryotic 18S rRNA gene (Euk_1391f–EukBr, (http://www.Earthmicrobiome.Org/emp-standard-protocols/18s/) were prepared and sequenced for each sample at the Australian Genome Research Facility (Melbourne, Australia) and the Ramaciotti Centre for Genomics (Sydney, Australia). The 16S and ITS amplicons were sequenced using 300 bp paired end sequencing, while 18S amplicon reads were generated using 150 bp paired end sequencing.

### Amplicon sequence analysis

#### 16S rRNA genes

The quality of all Illumina R1 and R2 reads was assessed visually using FastQC [29]. Generally, a significant drop in read quality was observed in the last 50–100 bp of R2 and the last 10 bp of R1. As many base pairs as possible were trimmed, while still leaving an overlap to allow reliable merging of R1 and R2 reads, as assessed manually after merging with FLASH [30]. The 5’ end of each R1 sequence was trimmed by 10 bp, and each R2 by 70 bp. Sequences were merged using FLASH [30]. Several hundred sequences were merged manually and the results compared to the FLASH merges to ensure merging efficacy. Once efficacy was confirmed, merged sequences were passed to the open reference Operational Taxonomic Unit (OTU) picking and assigning workflow.

Following merging, FASTA format sequences were extracted from FASTQ files. Sequences < 400 bp, or containing N or homopolymer runs of > 8 bp, were removed using MOTHUR (v1.34.1) [31]. The remaining sequences were passed to the open reference OTU picking and assigning workflow (described below).

#### 18S rRNA genes

Illumina R1 and R2 reads were both trimmed by 30 bp to remove primers and adaptors. The reads were merged using FLASH [30] as described for 16S rRNA above, and results compared to a random subsample of sequences merged by hand. Following merging, FASTA-formatted sequences were extracted from FASTQ files. Sequences < 100 bp, or containing N or homopolymer runs of > 8 bp, were removed as described above. The remaining sequences were then passed to the open reference OTU picking and assigning workflow.

### ITS regions of rRNA operons

Only R1 sequences were used for ITS regions. R1 included the ITS1 region, upon which our current workflow is based. ITS2 region reads (from R2 reads) are available on request. FASTA files were extracted from FASTQ files, and complete ITS1 regions were extracted using ITSx [32]. Partial ITS1 sequences and those not containing ITS1 were discarded. Sequences comprising full ITS1 regions were passed to the OTU picking and assigning workflow.

### Open OTU picking and assignment

Each of the four amplicons was submitted to the same workflow, separately, to pick OTUs and assign read abundance to a Sample-by-OTU matrix. This workflow followed a similar conceptual outline to that advocated in the QIIME open reference OTU picking pipeline [33], with the following differences: a) USEARCH 64 bit v8.0.1517 was employed directly; b) reference OTUs were not initially assigned via a round of closed reference picking, instead *de novo* OTUs were picked (OTUs were classified later); c) in order make compute time manageable for *de novo* picking, OTUs were initially picked on the numerically dominant sequences only (sequences with > 6 representatives across the full dataset); d) instead of randomly picking sequences that failed to be recruited to OTUs for subsequent clustering, all sequences with >2 representatives were used. USEARCH was primarily used for analysis, but other programs could be equally efficacious. The workflow can be summarised as follows:

1. Dereplicate sequences.
2. Sort sequences by abundance and keep sequences with > 6 representatives.
3. Cluster sequences into OTUs of ≥ 97 % similarity using UPARSE [34] and check for chimeras (outputs comprised both a representative OTU sequence file and a UPARSE file).
4. Cluster chimeric sequences to produce a representative sequences file for each OTU cluster (97 % similarity) [35] using the UPARSE output from (3) to obtain chimeric reads. The USEARCH “fast cluster” algorithm [34, 35] was used.
5. Concatenate de novo OTUs from (3) and chimeric OTUs from (4) into a single OTU FASTA mapping file.
6. Map reads in the original dataset of quality-checked sequences (1) against the output from (5) using the “usearch_global” function in USEARCH [34].
7. Split mapped reads (hits) from (6) into chimeric and non-chimeric output files.
8. Retrieve non-mapped reads (misses) from (6) from the original data to create a data set of non-mapped and non-chimeric reads, forming the basis of a second round of OTU picking.
9. Repeat the process from (2) with the non-mapped sequences from (8), with the number of required representatives per sequence at (3) reduced appropriately (e.g. from 6 to 2).
10. Concatenate the resultant USEARCH cluster files to create a final mapping file.
11. Convert the final mapping file to an OTU table.
12. Concatenate all representative OTU sequence files to produce final OTU representative set.
13. Identify OTUs using Green Genes (13-5) for bacteria and archaea; UNITE (v7.0) for fungi and SILVA (123) for eukaryotes. Classify MOTHUR’s implementation of the Wang classifier [36] at 60 % sequence similarity cut-off.
14. Create a final sample-by-OTU data matrix and taxonomy file by discarding sequences not identified as belonging to the correct lineage (i.e., bacteria, archaea, fungi, eukaryotes), unidentified at the phylum level, or having < 50 sequences across all samples in the database.

These final curation steps were guided by the inclusion of mock community samples (data not included) and reduced the number of OTUs considerably (e.g., bacterial OTUs from > 400,000 to < 90,000), while only removing < 1 % of the total sequences. It should be noted that these curation steps were performed for OTU table generation; raw FASTQ files of sequences (i.e. all sequences generated) are also available from the database.

## Database description

### BASE objectives and data usage

BASE is being developed to:

- Generate a comprehensive audit of Australian soil biodiversity;
- Assist bio-discovery to add to the known global diversity of key ecological groups;
- Model relationships between environmental parameters and microbial diversity;
- Examine the importance of microbes in generating ecological complexity, stability and resilience;
- Test broad biogeographical and evolutionary hypotheses regarding microbial evolution and plant–microbe co-evolution;
- Inform the restoration of soil communities as part of on-going broad-scale re-vegetation;
- Provide a baseline reference data set to examine the effects of land management;
- Inform the role of microbes in plant productivity, mineralogy and general soil health.

The BASE database [37] provides a rich source of microbial sequences and associated metadata for Australian soil ecosystems that can be used to further understanding of soil microbiological processes critical to ecosystem function and environmental health. The BASE project has sampled 902 sites and is continually expanding as new data become available. Although the number of potential biases that might influence data utility in any metagenomics/amplicon-based analysis (e.g. DNA extraction [38], PCR primer choice [39, 40], reagent contamination [41] etc.) is large, all samples were treated with the same protocols and therefore should all have the same biases. For microbiome characterisation we used the same protocols as those employed by the Earth Microbiome Project (EMP) [42] to ensure maximum compatibility with global data. To this end, the BASE project has also taken precautions to ensure that all procedural and analytical variables have been recorded, all samples were collected and transported according to the same method, and all DNA extractions and soil analyses were conducted by one of two facilities (Australian and Antarctic samples).

Many methods are available to analyse amplicon data; each having advantages and disadvantages. Indeed, it is often necessary to tailor the analysis to the specific question being addressed. The rationale behind amplicon data analysis for the BASE project was to provide a searchable framework for data exploration via our data portal, with sample-by-OTU matrices for most applications, and to ensure that raw data sources can be identified to allow future reanalysis if required.

All data collected by the project is publically available via the BASE data portal (Https://ccgapps.Com.Au/bpa-metadata/base/) which provides a searchable interface to explore BASE data, identify samples of interest and download data. The database contains biological, edaphic and other site-related data for each sample collected. The data may be interrogated for all data types (biological or non-biological), together or separately. For non-biological data comprising a single matrix of site-wise contextual data, empty cells indicate that no data is available for that sampling point, while a ‘sentry’ value of 0.0001 indicates values below the detection threshold for a particular assay. Actual detection limit values for each assay are displayed via a link on the contextual data page (Https://ccgapps.Com.Au/bpa-metadata/base/contextual/samplematrix). Columns on this page may be sorted numerically or alphabetically.

**Fig. 3:**
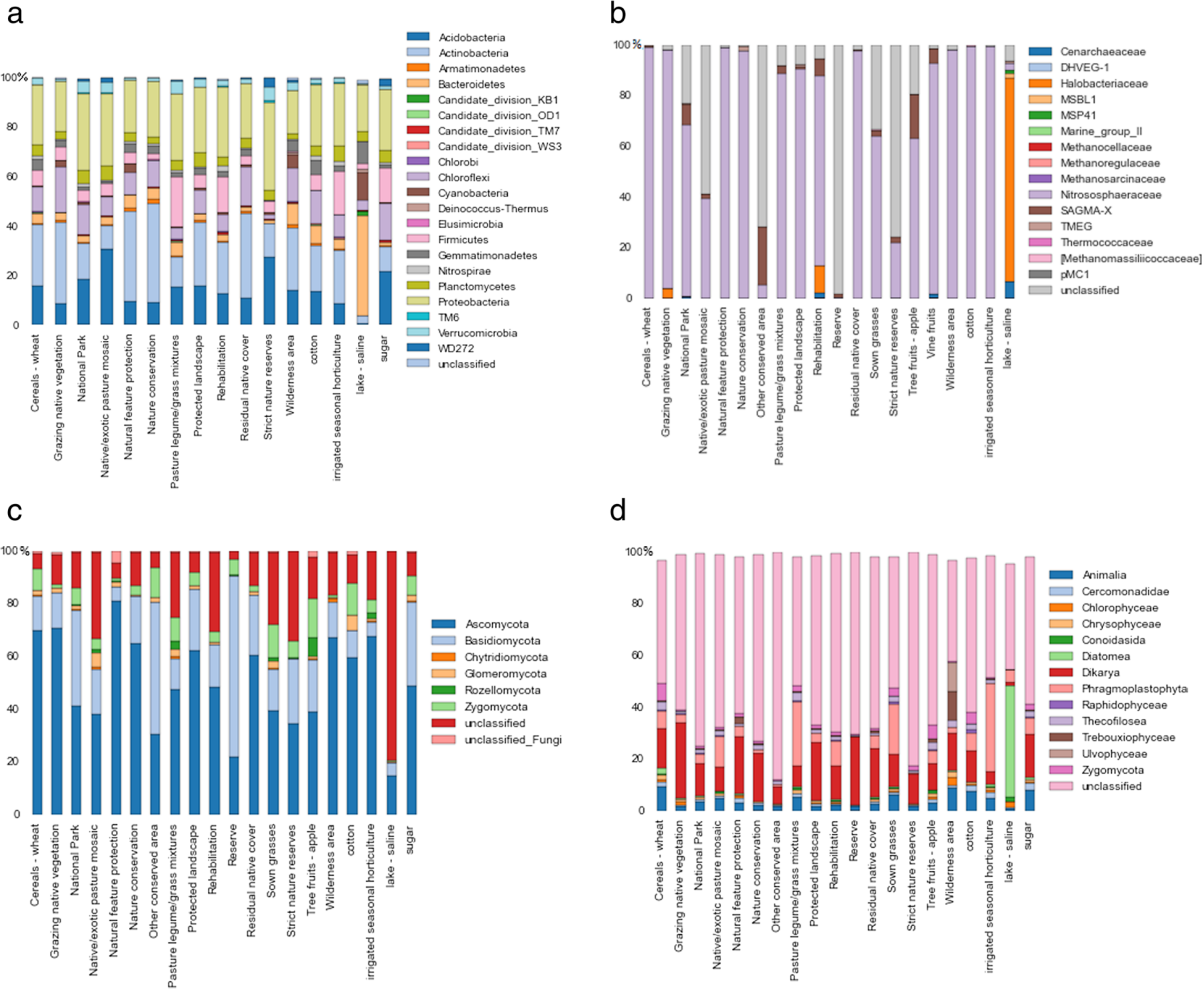
Microbial diversity under different land-use categories sampled in BASE. **a** Bacterial phyla comprising > 1 % of total bacterial 16S rRNA gene amplicons; **b** archaeal families comprising > 1 % of total archaeal 16S rRNA gene amplicons; **c** fungal phyla comprising > 1 % of total fungal ITS1 region amplicons; and **d** eukaryotic phyla comprising > 1 % of 18S rRNA gene amplicons. All abundances are expressed in % of the total read number for each group, and land-use categories refer to land-use categories as described in the Australian land use and management classification (http://www.agriculture.gov.au/abares/aclump/land-use/alum-classification-version-7-may-2010)

We aim to include a minimum of 20,000 sequences in the BASE database for each sample and amplicon. While previous work has shown that around 2000 sequences are enough to preserve between sample (treatment) differences [43], this number of sequences does not saturate coverage curves in most environments. We have therefore sought to produce as many sequences as resources allow. Most samples sequenced thus far exceed this number, and those falling below this threshold are being re-sequenced to increase the number of sequences per sample to > 20,000. Details of sequencing outputs for each amplicon are contained in Table 2 and diversity for each land-use category is presented in Fig. 3. Biological data are available as both processed and raw sequence data for all samples or subsets, as defined by database searches. Processed data comprises sample-by-OTU tables for the samples/taxonomies of interest, and a FASTA-formatted sequence file containing representative sequences for all OTUs. These are provided separately for each amplicon. Data are also provided as raw Illumina paired end sequence files for each sample. These data can be searched and downloaded via the database (Https://ccgapps.Com.Au/bpa-metadata/base/search). This search facility allows users to identify samples of interest based on amplicon taxonomy and/or site contextual data.

**Table 2:** Details of sequencing outputs for each amplicon

Amplicon	Bacteria	Archaea	Eukaryote	Fungi
Total reads^a^	67578131	99533527	65086341	86322772
Mean per sample	74837 ± 59400	97009 ± 56696	74153 ± 58634	103504 ± 131838
OTU Richness	85596	5421	21552	43708
% classified^b^	72 %	22 %	40 %	69 %

^a^ Total number of sequences after all QC and processing

^b^ % classified to family level (>60 % probability) against Green Genes for Bacteria and Archaea, UNITE for Fungi and SILVA for Eukaryotes

The database portal also contains a sample distribution map showing sample sites and providing site-specific information in the context of site geographic position (Https://ccgapps.Com.Au/bpa-metadata/base/contextual/sites), contextual data tables for all sites (https://ccgapps.Com.Au/bpa-metadata/base/contextual/samplematrix), all BASE project related methods, and lists of all currently available amplicon and metagenomic samples.

## Sampling design

The sampling protocols for the BASE project were developed with several constraints in mind:

1. For every physical sample sequenced, soil contextual data are required.
2. The more contextual data variables collected, the greater the requirement for physical sample.
3. A soil sample at any size/scale appropriate for both sequence and contextual data generation is necessarily a composite sample. The sample may be as small as possible to give the required amount of soil for sequencing and contextual data generation, but the sample is nonetheless required to be well mixed/homogeneous.
4. Single point samples are destructive and do not easily facilitate temporal monitoring.

The sampling scheme as described above (nine samples over a 25 m × 25 m quadrat, homogenised into a single sample) was chosen because it generated sufficient physical sample material for sequencing (i.e. enough DNA for amplicon and shotgun library generation), chemical and physical analyses, and sample archiving; easily facilitated temporal sampling points, allowed integration of microbial data with landscape elements and other biological data collected at similar scales; and is easily implemented by unskilled practitioners. This sampling scheme provides broad benefits for increasing our knowledge of soil biomes at a continental, regional and local scale, although is not suitable to answer questions relating to scales less than 25 × 25 m. Indeed, the sampling scheme is a compromise between available resources and the competing uses for which data are generated.

## Data visualisation

The current visualisation tools available via BASE are being developed in an on-going collaboration with the Atlas of Living Australia (Http://www.Ala.Org.Au) and provide a platform to visualise BASE-derived microbial diversity data in the context of other Australian diversity and environmental data [44]. Currently, analysed BASE OTU and contextual data are available via a persistent instance of ALA’s sandbox tool (Http://base.Ala.Org.Au/datacheck/datasets). This resource is linked from the BASE data portal and the BASE project description pages, and allows users to both visualise BASE site-related data on geographic maps, as text records, plot charts showing sample attribute distributions, and to intersect BASE collected data with ALA provided environmental, occurrence, diversity and climate data. Five datasets are currently available (site contextual data and data for the four BASE amplicons targeting bacteria, archaea, fungi and eukaryotes).

## Current uses

Data from the project has helped to address questions about the impacts of agricultural management practices; for example, the use of nitrogen fertilizer on soil microbiomes in sugar cane production in coastal Queensland. Previous work demonstrated that nitrogen applied to soils is diminished within 2–3 months, although the crop requires nitrogen from soil for at least 6 months. Soil microbes convert fertilizer into leachable and gaseous forms of nitrogen, including the greenhouse gas nitrous oxide, which results in considerable inefficiencies and environmental penalties [45]. Metagenomic data confirmed elevated abundances of genes involved in nitrification and denitrification following fertilizer application, corroborating the inference that agricultural soil microbiomes are attuned to scavenging nitrogen for their own energy metabolism [46]. The study demonstrated that low rates of nitrogen fertilizer application over several years did not increase the abundance of diazotrophic microbes and Nif genes in soil or in association with sugarcane roots, indicating that active manipulation of microbial communities may be required to boost biological nitrogen fixation [35]. Amplicon data also indicated a small yet significant effect of fertilizer application on bacterial [46] and fungal community composition [47]. This approach also identified the microbes that were enriched in the rhizosphere and roots, allowing subsequent tests as to whether beneficial or detrimental microbes are prevalent, and which microbes are potential candidates for formulating bioinocula with plant-growth-enhancing rhizobacteria [48].

In other applications, BASE data are used to model microbial community spatial turnover, the effect of edaphic and climate factors on microbial community structure, to elucidate microbial community assembly and maintenance drivers at the continental scale, and to inform the most efficacious target sites for future sampling efforts. For example, at various points in the development of the database survey gap analysis methods [49, 50] were used to identify Australian soils that may contain diversity not yet captured in the database [51, 52].

## BASE: future outlook

The BASE database is an evolving, continuously improving resource, both in terms of the number of samples included in the database, and the way in which the database may be utilised. We will provide updates on advances and tool development on the project’s online documentation pages.

Despite providing useful data exploration resources, the present BASE visualisation tools available via ALA are limited to presence/occurrence of organisms (rather than abundance). Furthermore, they are linked to current taxonomy/classifications and cannot directly compare two or more sites. Through on-going collaboration with the ALA, BASE is developing methods to address these shortcomings, including incorporating abundance data. BASE data will make use of the ALA phylogeny-based interrogative visualisation tools (Http://phylolink.Ala.Org.Au) [53]. ALA Phylolink will allow users to view Australian soil microbial diversity in terms of phylogeny, in addition to taxonomy, through the incorporation of collapsible phylogenetic trees. These trees will interact with Australian diversity map layers to allow users to build powerful visualisations of soil microbial and other soil/diversity data, bringing the BASE data set into context with other Australian biodiversity data (e.g., mapped soil edaphic properties, plant and animal diversity etc.). We are developing the capability to compare and graph differences between two or more samples. Finally, we anticipate that the current segregation of species occurrence data by domain/kingdom and environment (e.g., soil, aquatic, marine) will not persist, and that all biodiversity and site contextual data will be combined into an integrated system. This will allow integrative ecological approaches to be pursued. Incorporation of the BASE data set into wider Australian ecological data sets, as used by ALA, for example, will be an important step in achieving in this.

The priorities for additional sampling include the incorporation of a temporal aspect by re-sampling sites, the inclusion of more examples/replicates of each land-use and management strategy within land-use, particularly for agricultural samples, and samples identified from survey gap analysis as likely harbouring uncaptured diversity. As well as directly generating further samples through this initiative, we aim to accommodate independently generated Australian microbial diversity data within the database.

Finally, the BASE database currently comprises primarily amplicon-derived data from all three domains of microbial life. However, this will be expanded to include amplicon-free metagenomic sequencing from approximately 500 sites (0–0.1 m depth) (Https://ccgapps.Com.Au/bpa-metadata/base/information). These sites have been chosen to maximise geographic spread, and diversity of land-use, soil type and aboveground ecosystem. Initially, metagenomics data have been made available via the European Bioinformatics Institute (EBI) metagenomics portal (Https://www.Ebi.Ac.Uk/metagenomics/) and can be found by searching “BASE” in EBI metagenomics projects. Data are uploaded to EBI as they become available (12 sites available so far). Once the ~500 samples have been sequenced (expected by May 2016), a trait-by-sample table will be added to the BASE data portal search facility, where “trait” refers a functional gene metabolic pathway.

## Summary

The BASE project represents the first database of Australian soil microbial diversity that has been developed in the context of an open data/open access framework. It will continue to grow as more samples are sequenced and added, and as the community of users grows. As the BASE data set expands it will become further linked with other biodiversity exploration efforts (global microbial, plant, animal, marine, etc.) and environmental data sets. Immediate priorities include additional sampling to improve the representation of Australia’s climate, soil, ecological and land-use diversity, and to incorporate a temporal dimension by repeat sampling of selected sites. Database design elements, combined with these additional priorities, will allow the BASE project to evolve as a valuable tool to document an often overlooked component of biodiversity and address pressing questions regarding microbially mediated processes essential to sustained soil function and associated ecosystem services.

## Availability of supporting data

The dataset supporting this article is available in the BioPlatforms Australia project’s data portal (Https://ccgapps.Com.Au/bpa-metadata/base/), DOI 10.4227/71/561c9bc670099 [37]. All raw data has been deposited in the Sequence Read Archive (SRA) under the Bioproject ID PRJNA317932. Information on all SRA accessions related to this dataset can also be found at (Https://downloads.Bioplatforms.Com/metadata/base/amplicon/amplicons). All OUT pipelines can be found at (http://www.Bioplatforms.Com/soil-biodiversity/) under “BASE protocols and Procedures”.

## Abbreviations

AAD: Australian Antarctic Division
AGRF: Australian Genome Research Facility
ALA: Atlas of Living Australia
BASE: Biomes of Australian Soil Environments
OTU: Operational Taxonomic Unit
