# Erratum to: Introducing BASE: the Biomes of Australian Soil Environments soil microbial diversity database

**DOI:** 10.1093/gigascience/gix021

**Published:** 2017-05-08

**Authors:** Andrew Bissett, Anna Fitzgerald, Leon Court, Thys Meintjes, Pauline M. Mele, Frank Reith, Paul G. Dennis, Martin F. Breed, Belinda Brown, Mark V. Brown, Joel Brugger, Margaret Byrne, Stefan Caddy-Retalic, Bernie Carmody, David J. Coates, Carolina Correa, Belinda C. Ferrari, Vadakattu V. S. R. Gupta, Kelly Hamonts, Asha Haslem, Philip Hugenholtz, Mirko Karan, Jason Koval, Andrew J. Lowe, Stuart Macdonald, Leanne McGrath, David Martin, Matt Morgan, Kristin I. North, Chanyarat Paungfoo-Lonhienne, Elise Pendall, Lori Phillips, Rebecca Pirzl, Jeff R. Powell, Mark A. Ragan, Susanne Schmidt, Nicole Seymour, Ian Snape, John R. Stephen, Matthew Stevens, Matt Tinning, Kristen Williams, Yun Kit Yeoh, Carla M. Zammit, Andrew Young



**Correction:**

In the article ‘Introducing BASE: the Biomes of Australian Soil Environments soil microbial diversity database *GigaScience 2016 5:21*’ [1] the authorship list should have included Leon Court, who was responsible for sample collection and preparation, sampling design and sequencing method design. The authors regret this omission.
